# Notes and Notices

**Published:** 1899-06

**Authors:** 


### NOTES AND NOTICES.

See Speer's Chateau wine cellar of New Jersey vineyards. Read all about it and about the unfermented Grape Juice.

### PICRIC ACID IN THE TREATMENT OF BURNS.

About six months ago I began the use of picric acid in the treatment of this class of injuries. The results were uniformly so satisfactory that I now depend wholly on it in burns and scalds of the first and second degree. This form of treatment has been in use in France for some years, but not in America. I have always used what is known as a saturated solution, which consists of ninety grains of salt dissolved in three ounces of alcohol, and this diluted with one quart of water. The clothing of the part having been removed, the burnt surface is cleansed with some of the solution and absorbent cotton. Blisters are opened and serum allowed to escape, but the epithelium covering preserved. If area involved is extensive the whole surface may be bathed in the solution and strips of sterilized gauze soaked and applied to entirely cover it. Over this a layer of absorbent is placed and all held in place by a light bandage. The dressing soon dries and should be allowed to remain three or four days before trying to remove it when it should be thoroughly moistened and carefully removed, as it adheres to the burnt surface very closely. The second dressing is applied as the first and is allowed to remain a week. Among its disadvantages is the staining of the hands and bed-clothes but a solution of boric acid will remove this.—*Dr. A. L. Blackwood, in The Clinique.*

### PORT GRAPE WINE FOR THE SICK.

"We can confidently recommend Speer's Port Grape Wine, a superior article of wine for the sick and debilitated."—*Medical Review.*

No brandy is better than Speer's * * * *Climax* of 1878.

### REMOVED.

Munson & Co., Western Homœopathic Pharmacy, established 1868, W. F. Bockstruck, proprietor, have removed to their grand new store, Stifel building, 908 Pine street, St. Louis, Mo. The largest selection of new goods, new books, new medicines. The purity of our medicines is warranted and our prices are the lowest.

### NEW JERSEY GRAPE JUICE SENT TO EUROPE.

Mr. Speer, of New Jersey, has a reputation extending over the world as being a reliable producer of Oporto Grape Juice and Port Wine. They are ordered by families in Dresden, London and Paris for their superior virtues.

### BROKEN DOSES.

A very subdued looking boy of about 13 years, with a long scratch on his nose and an air of general dejection, came to his teacher and handed her a note, before taking his seat and becoming deeply absorbed in his book. The note read as follows: "Miss B., please excuse James for not being thare yesterday. He played trooant, but I guess you don't need to lick him for it, as the boy he played trooant with an' him fell out an' the boy licked him, an' a man they sassed caught him an' licked him, an' the driver of the sled they hung onto licked him also. Then his pa licked him, an' I had to give him another one for sassing me for telling his pa, so you need not lick him until next time. I guess he thinks he better keep in school hereafter."—*Leon (Kan.) Indicator.*

